# Supplementary material for: FTO deficiency in older livers exacerbates ferroptosis during ischaemia/reperfusion injury by upregulating ACSL4 and TFRC
Source: Nat Commun. 2024 Jun 4;15:4760. doi: 10.1038/s41467-024-49202-3 (PMC11150474; doi:10.1038/s41467-024-49202-3)
Supplement: Supplementary file 1 — Supplementary Information [file 41467_2024_49202_MOESM1_ESM.pdf]

Supporting information

FTO deficiency in older livers exacerbates ferroptosis during ischaemia/reperfusion injury by upregulating ACSL4 and TFRC

Rong Li, Xijing Yan, Cuicui Xiao, Tingting Wang, Xuejiao Li, Zhongying Hu, Jinliang Liang, Jiebin Zhang, Jianye Cai, Xin Sui, Qiuli Liu, Manli wu, Jiaqi Xiao, Haitian Chen, Yasong Liu, Chenhao Jiang, Guo Lv, Guihua Chen, Yingcai Zhang, Jia Yao, Jun Zheng, Yang Yang

Table of Contents

Supplementary figures……………………………………………………………….……1

Supplementary tables…………………………………………………………………18

Supplementary Figures

Fig. S1

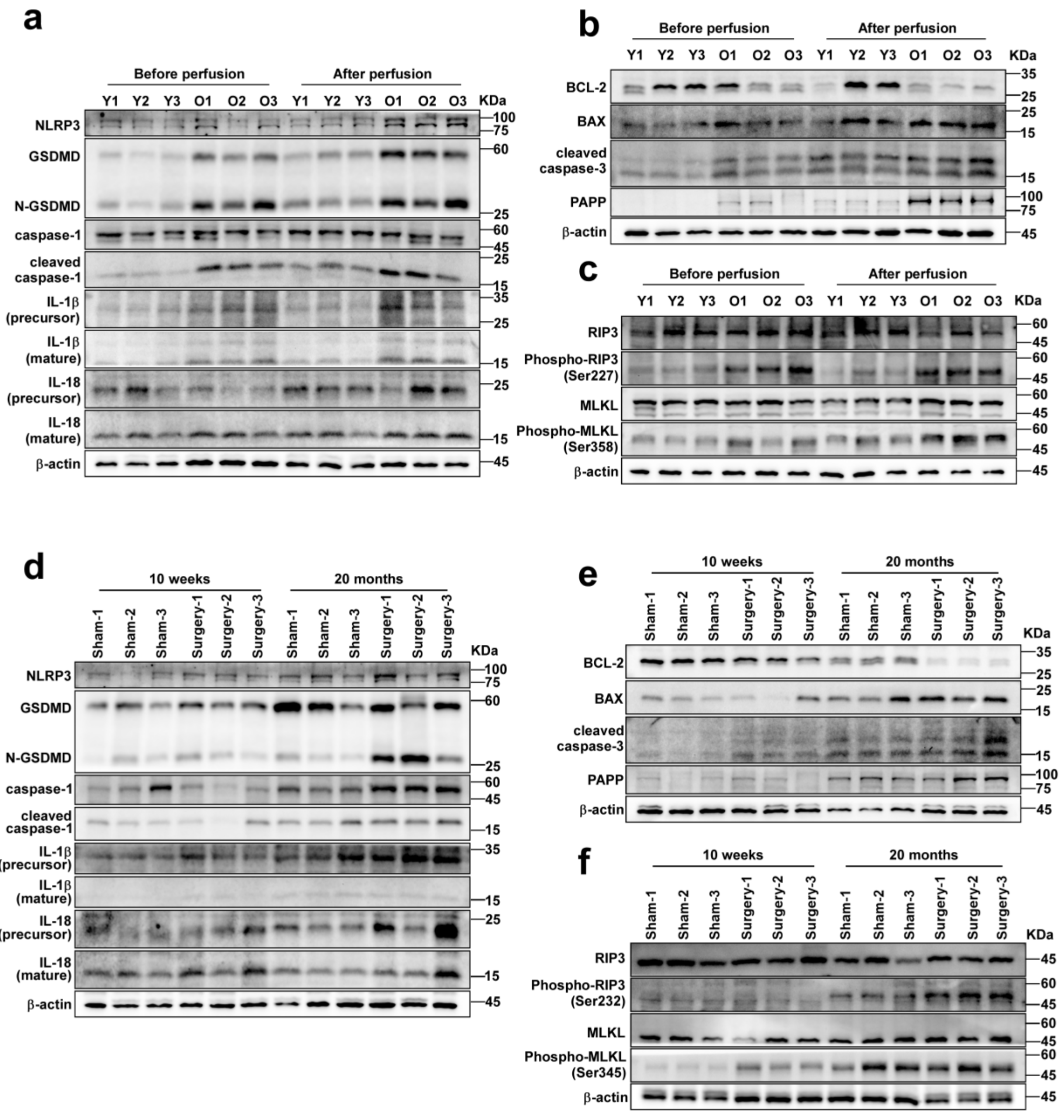

Fig. S1 A brief overview of multiple types of cell death in young and aged HIR. (a-c) The expression of key proteins related to pyroptosis (a), apoptosis (b) and necroptosis (c) in young/aged human liver tissues before or after refusion. **(d-f)** The expression of key proteins related to pyroptosis (d), apoptosis (e) and necroptosis (f) in mouse liver tissues suffered from IRI. Three independent biological human samples and mice samples, source data are provided as a Source Data file.

Fig. S2

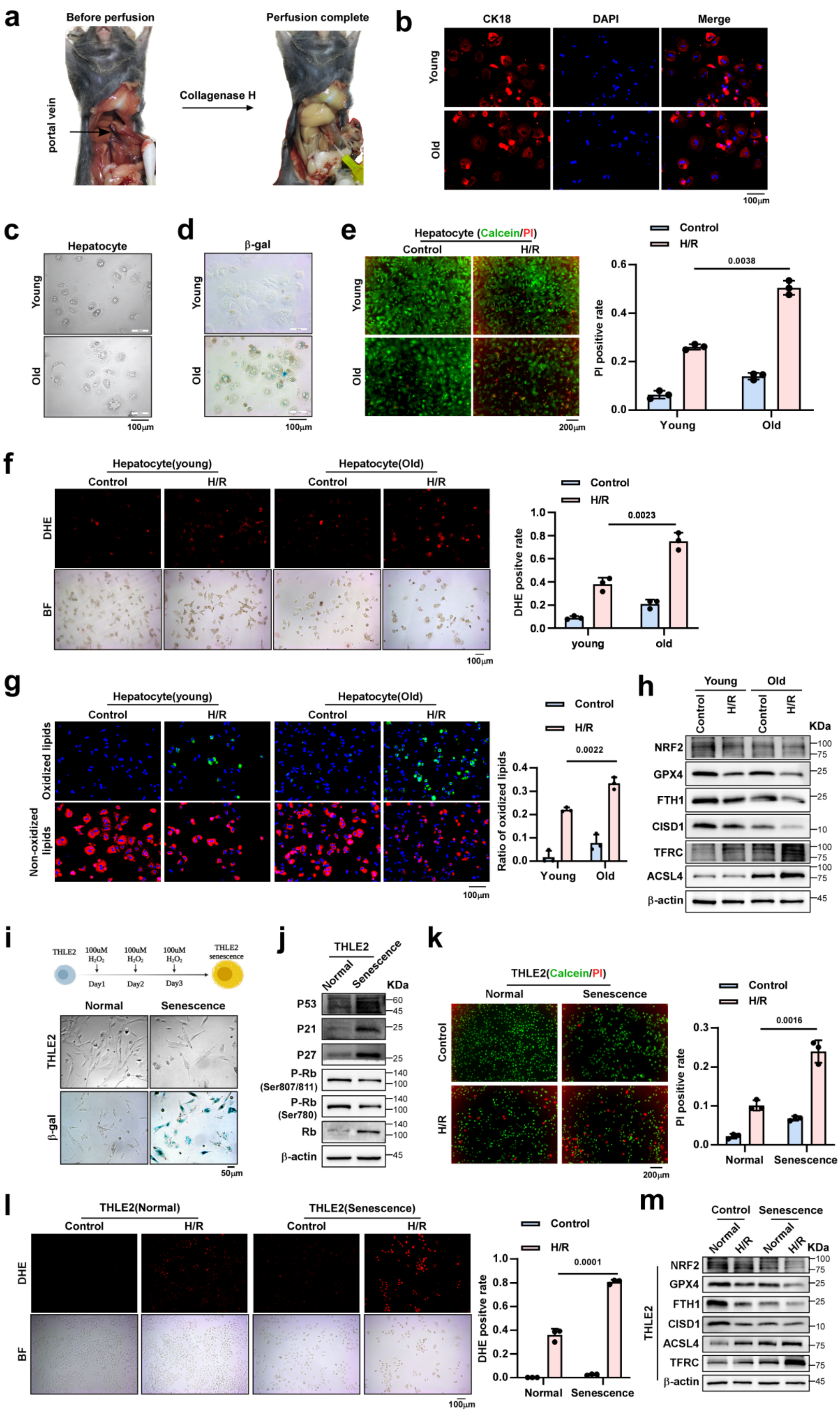
a
Before perfusion
Perfusion complete
Collagenase H
portal vein
b
CK18
DAPI
Merge
Young
Old
100μm
c
Hepatocyte
d
β-gal
e
Hepatocyte (Calcein/PI)
Control
H/R
PI positive rate
0.0038
f
Hepatocyte(young)
Hepatocyte(Old)
DHE
BF
DHE positve rate
0.0023
g
Oxidized lipids
Non-oxidized lipids
Ratio of oxidized lipids
0.0022
h
NRF2
GPX4
FTH1
CISD1
TFRC
ACSL4
β-actin
KDa
i
THLE2
100uM H2O2
Day1
Day2
Day3
THLE2 senescence
Normal
Senescence
50μm
j
P53
P21
P27
P-Rb (Ser807/811)
P-Rb (Ser780)
Rb
k
THLE2(Calcein/PI)
0.0016
l
THLE2(Normal)
THLE2(Senescence)
0.0001
m
200μm

Fig. S2 Aged hepatocytes are more prone to injury and are characterized by increased ferroptosis during H/R. (**a**) Illustration of the extraction of mouse primary hepatocytes. (**b**) Validation of mouse primary hepatocytes with CK18 staining (magnification, 200×). (**c**) Morphology of young and aged primary hepatocytes (magnification, 200×). (**d**) β-gal staining of young and aged primary hepatocytes (magnification, 200×). (**e-g**) Representative images and relative quantification of Calcein-acetoxymethyl (Calcein-AM) / propidium iodide (PI) double staining assays (magnification, 100×), DHE staining (magnification, 100×) and C11 BODIPY staining (magnification, 200×) to examine cell death, ROS accumulation and lipid peroxidation in young and aged hepatocytes during H/R, two-tailed *t*-test. (**h**) Western blotting showed the expression of key proteins related to ferroptosis in hepatocytes in different groups during H/R. (**i**) Illustration of the induction of THLE2 cell senescence (upper) and the relative validation (lower) via cell morphology and β-gal staining (magnification, 200×). (**j**) The validation of cell senescence with western blotting. (**k-l**) Representative images and relative quantification of Calcein-AM / PI double staining assays (magnification, 100×) and DHE staining (magnification, 100×) in THLE2 cells in different groups during H/R, two-tailed *t*-test. (**m**) The expression of key proteins related to ferroptosis in THLE2 cells in different groups during H/R via western blotting. Statistic data are presented as the mean ± SD, error bars represent the means of three independent experiments. $P < 0.05$ was considered statistically significant, source data are provided as a Source Data file. Figure S2i (upper) created with BioRender.com released under a Creative Commons Attribution-NonCommercial-NoDerivs 4.0

International license https://creativecommons.org/licenses/by-nc-nd/4.0/deed.en.

Fig. S3

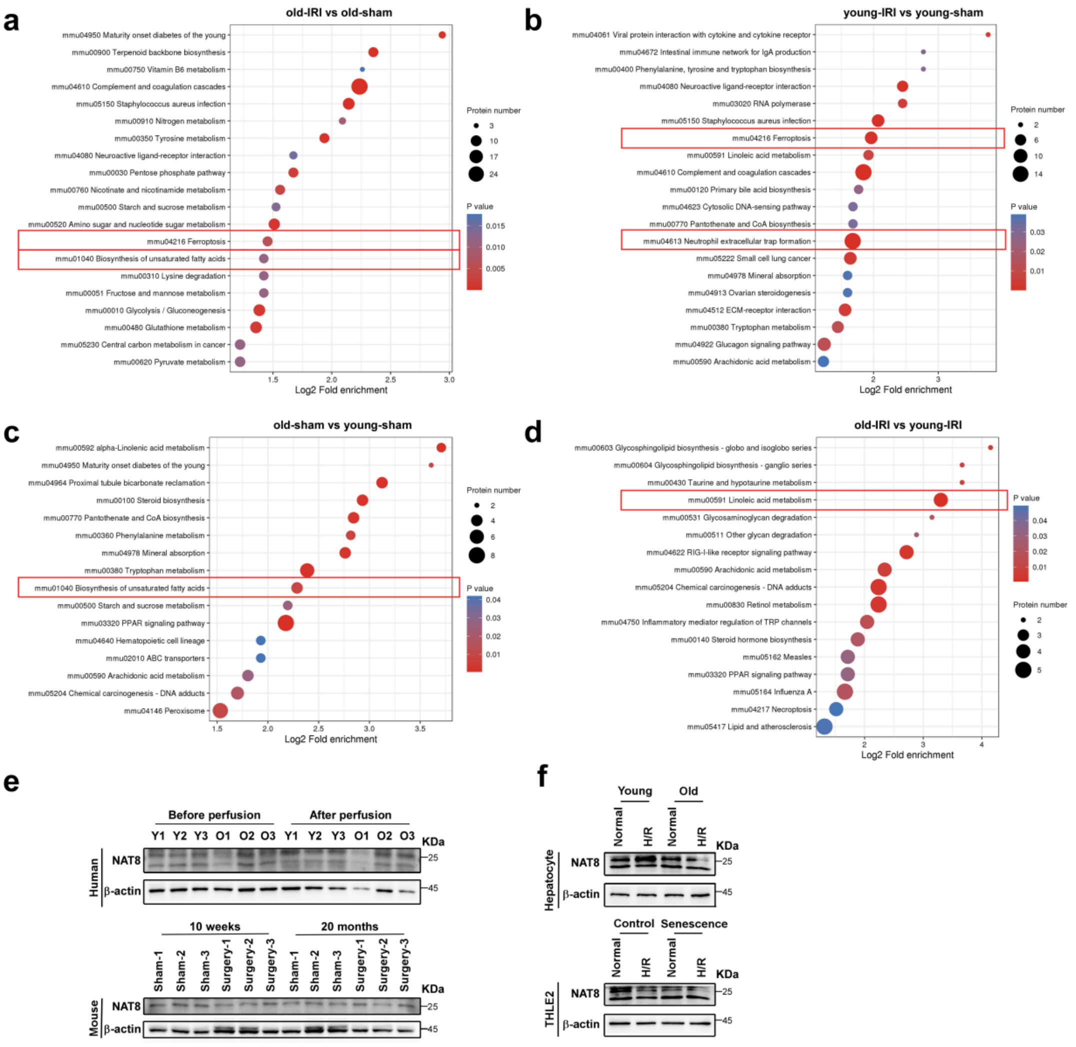

Fig. S3 Functional enrichment analysis and validation of differential proteins in young and aged HIRI. (**a**) Bubble chart showing the enriched signaling pathways in "old-IRI" vs "old-sham". (**b**) Bubble chart showing the enriched signaling pathways in "young-IRI" vs "young-sham". (**c**) Bubble chart showing the enriched signaling pathways in "old-sham" vs "young-sham". (**d**) Bubble chart showing the enriched signaling pathways in "old-IRI" vs "young-IRI". (**e-f**) Western blotting showing the

expression of NAT8 in liver tissues and hepatocytes from mice of different ages during IR, three independent biological human samples or mice samples and three independent cell experiments. Source data are provided as a Source Data file.

Fig. S4

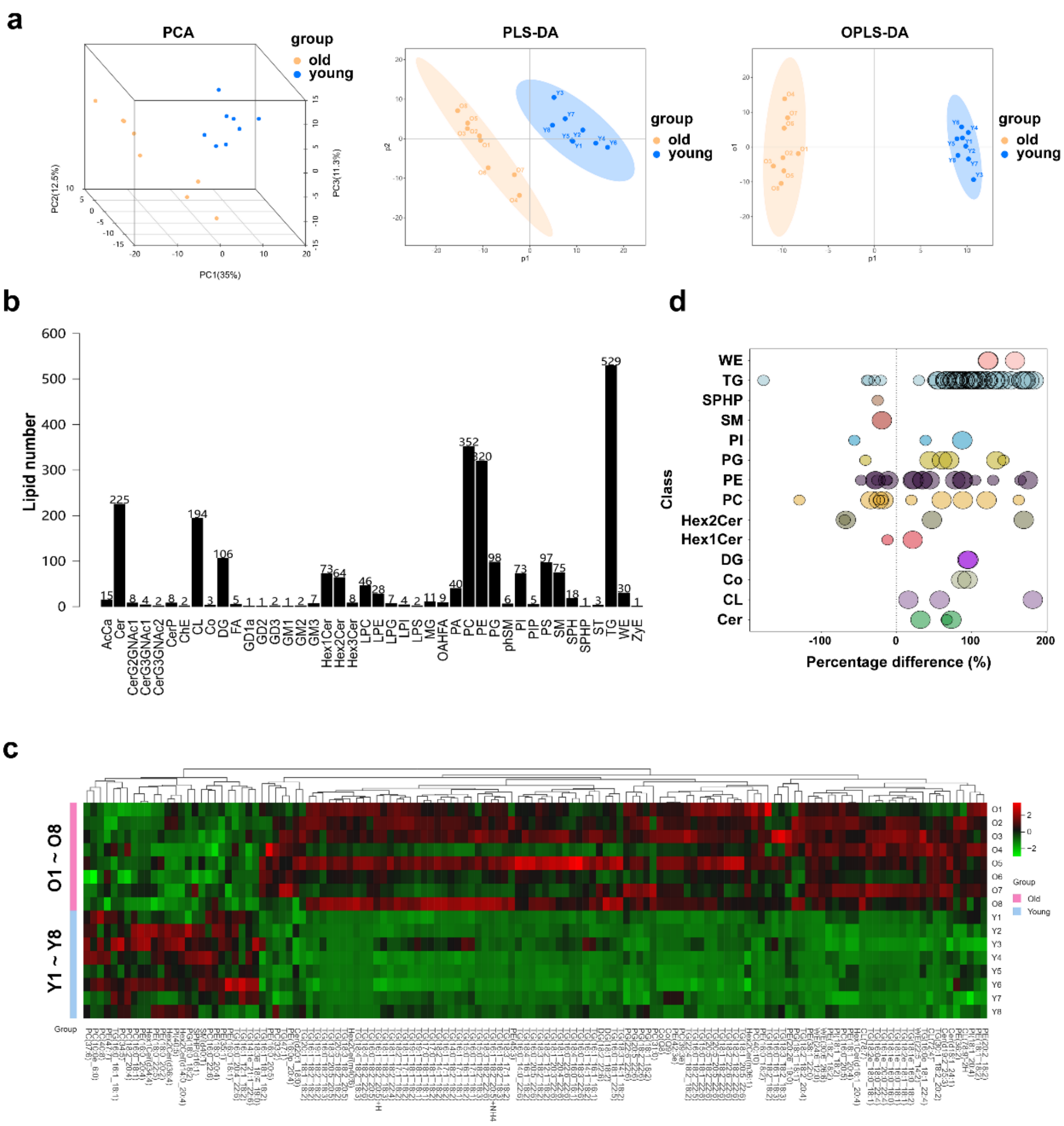

Fig. S4 The lipidomics profile of young and aged liver tissue. (**a**) PCA, PLS-DA, and OPLS-DA showed both intragroup repeatability and intergroup variability. (**b**) Bar chat showing the overview of lipid classes and related lipid species' numbers detected

in young and old liver tissues. (**c**) Heatmap showing differentially expressed lipid species in the indicated groups. (**d**) Bubble chats showing the top 14 lipid classes with the most significant differences in young and aged livers.

Fig. S5

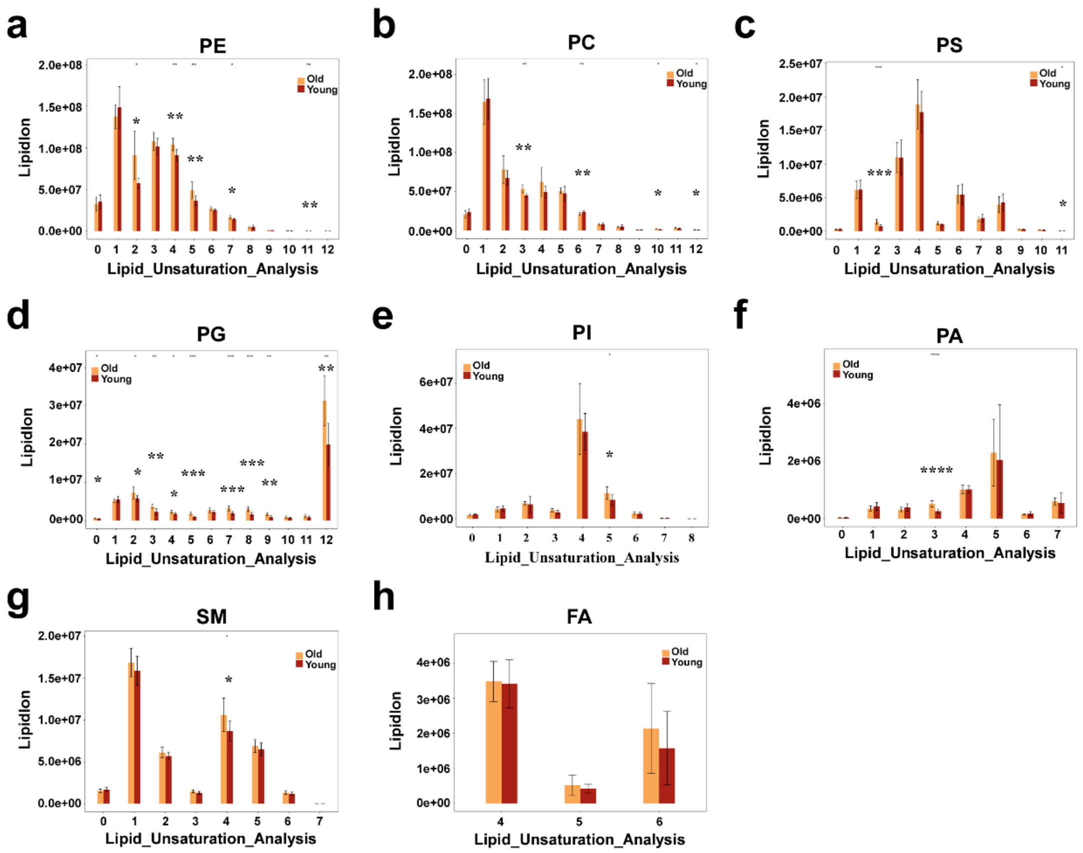

Fig. S5 The differential analysis of critical esterified lipids between young and aged liver tissue. (**a-h**) Bar charts showing the lipid unsaturation analysis of indicated esterified lipids, including phosphatidylethanolamine (PE), phosphatidylserine (PS), phosphatidylcholine (PC), phosphatidylglycerol (PG), phosphatidylinositol (PI), phosphatidic acid (PA), sphingomyelin (SM) and Fatty acid (FA). Data are presented as the mean ± SD, the differential analysis of critical esterified lipids was using two-

tailed *t*-test, *P < 0.05, **P < 0.01, ***P < 0.001, ****P < 0.0001. The complete lipid composition data are provided in the Source data file.

Fig. S6

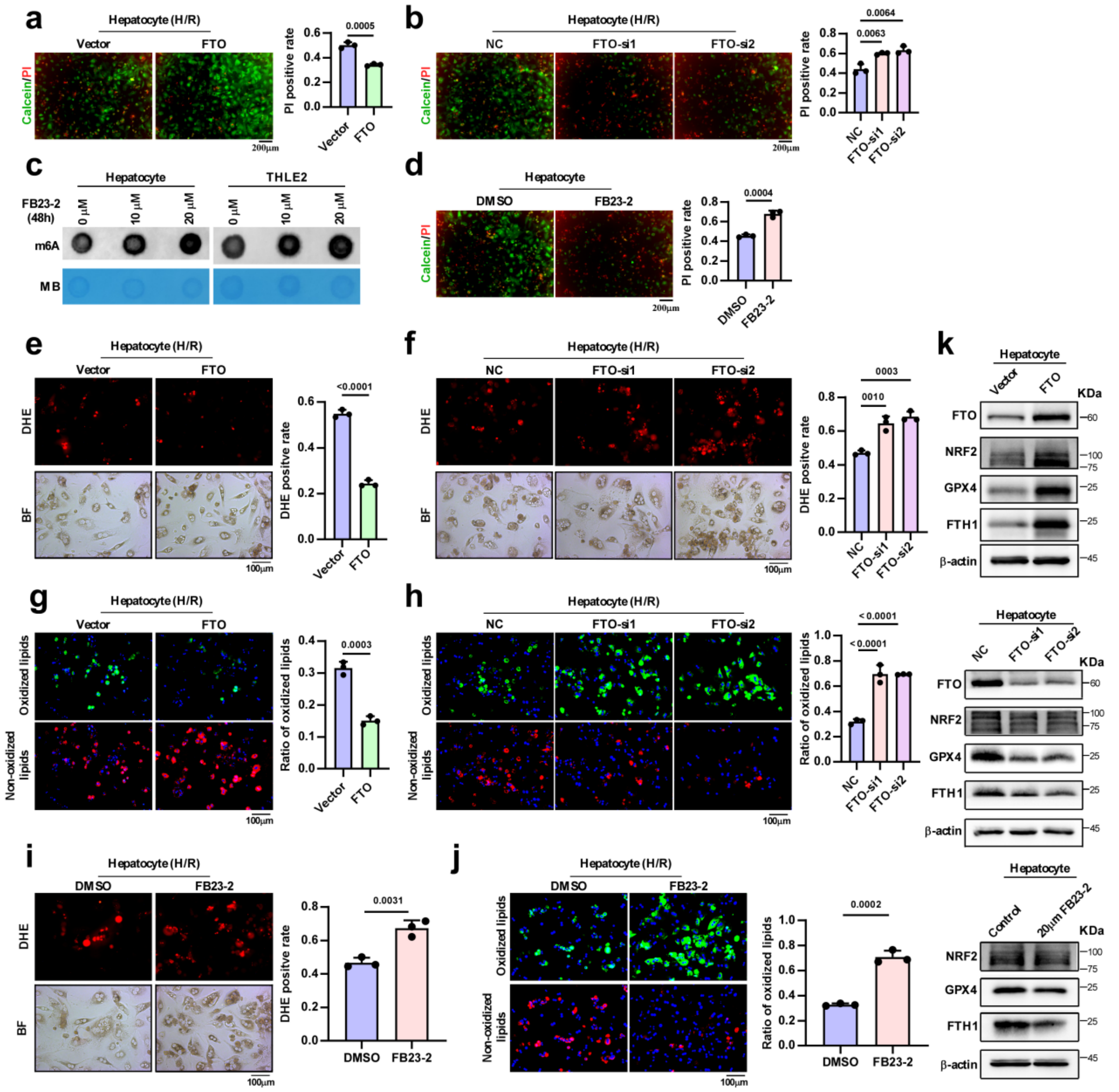

Fig. S6 FTO mitigates aged HIRI by decreasing ferroptosis (primary hepatocytes). (**a-b**) Representative images and relative quantification of Calcein-AM / PI double staining assays (magnification, 100×) in primary hepatocytes in different groups during H/R, two-tailed *t*-test. (**c**) Validation of the FB23-2-mediated inhibition of FTO

demethylase activity via dot blotting assay. (**d**) Representative images and relative quantification of Calcein-AM / PI double staining assays (magnification, 100×) in primary hepatocytes treating with FB23-2. (**e-j**) Representative images and relative quantification of DHE staining (magnification, 200×) and C11 BODIPY staining (magnification, 200×) of primary hepatocytes during H/R after different treatments, two-tailed *t*-test. (**k**) Western blotting showed the expression of primary hepatocytes in different groups during H/R. Statistic data are presented as the mean ± SD, error bars represent the means of three independent experiments. $P < 0.05$ was considered statistically significant. NS, no significance, source data are provided as a Source Data file.

Fig. S7

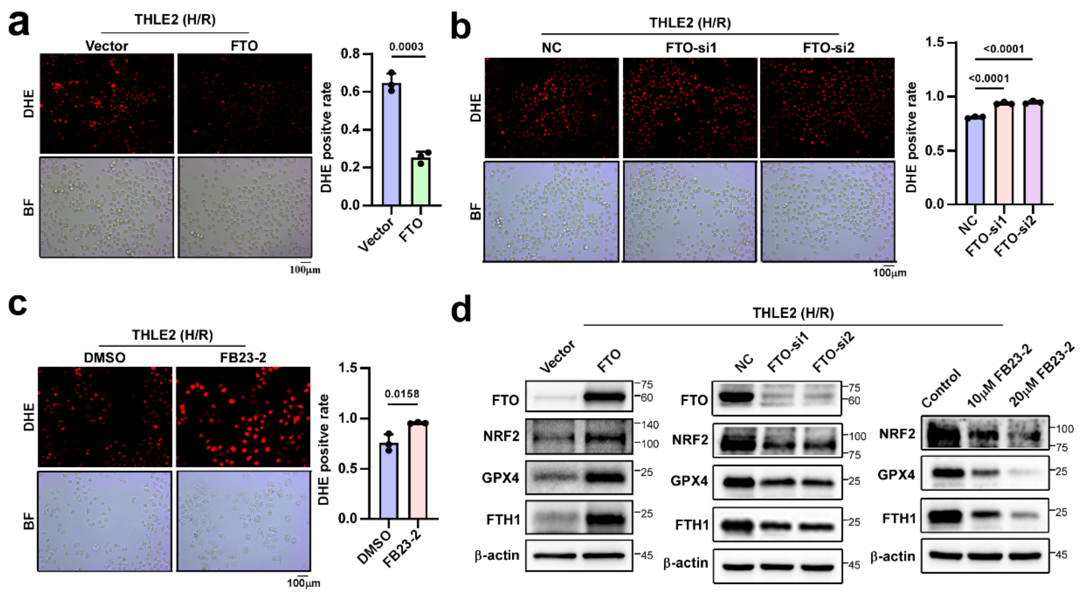

Fig. S7 FTO inhibits ferroptosis of senescent THLE2 during H/R. (a-c) Representative images and relative quantification of DHE staining (magnification, 100×) in senescent THLE2 cells in different groups during H/R. two-tailed *t*-test. (**d**)

The expression of key proteins related to ferroptosis in senescent THLE2 cells after different treatments via western blotting. Statistic data are presented as the mean ± SD, error bars represent the means of three independent experiments. P < 0.05 was considered statistically significant, source data are provided as a Source Data file.

Fig. S8

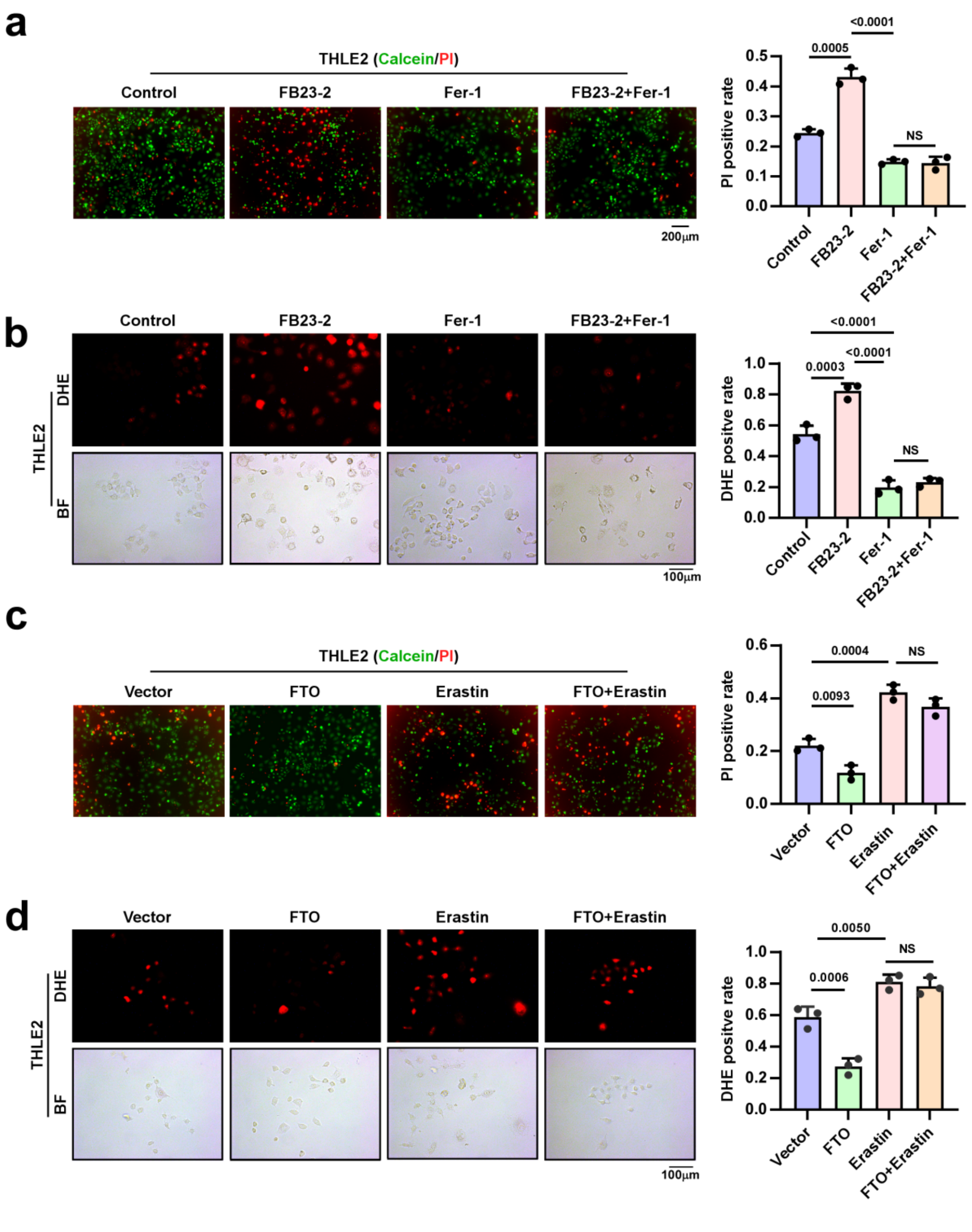

Fig. S8 FTO ameliorates hepatocellular damage during H/R depending on its regulation of ferroptosis. (**a-b**) Representative images and relative quantification of Calcein-AM / PI double staining assays (magnification, 100×) and DHE staining (magnification, 200×) to evaluate the effects of inhibiting ferroptosis on the FB23-2-mediated increase in cell death and ROS accumulation in senescent THLE2 cells during H/R, one-way ANOVA followed by multiple comparisons. (**c-d**) Representative images and relative quantification of Calcein-AM / PI double staining assays (magnification, 100×) and DHE staining (magnification, 200×) to evaluate the effects of ferroptosis induction on the FTO-mediated decrease in cell death and ROS accumulation in senescent THLE2 cells during H/R, one-way ANOVA followed by multiple comparisons. Statistic data are presented as the mean ± SD, error bars represent the means of three independent experiments. P < 0.05 was considered statistically significant. NS, no significance, source data are provided as a Source Data file.

Fig. S9

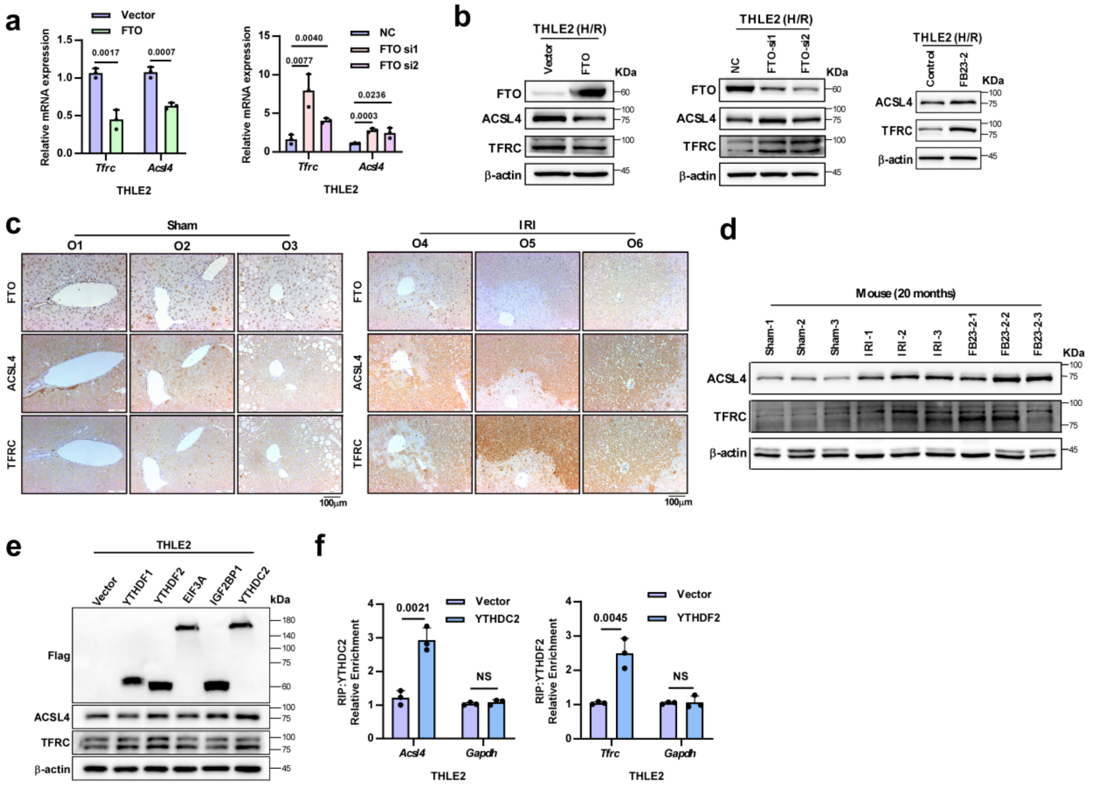

Fig. S9 FTO inhibits the expression of ACSL4 and TFRC. (**a-b**) Effects of FTO on the expression of ACSL4 and TFRC in senescent THLE2 cells via RT–qPCR and western blotting. RT–qPCR, two-tailed *t*-test. (**c**) Representative images of IHC staining (magnification, 200×) for FTO, ACSL4, and TFRC in the same set of consecutive aged liver tissue slices to evaluate the correlation between the expression of FTO and its targets. (**d**) The expression of ACSL4 and TFRC in aged liver tissues in different groups. (**c-d**) Three independent biological mice samples. (**e**) Effects of different m6A readers on the expression levels on ACSL4 and TFRC. (**f**) RIP assays showing the enrichment of *Acsl4* mRNA by YTHDC2 (left) and the enrichment of *Tfrc* mRNA by YTHDF2 (right), two-tailed *t*-test. Statistic data are presented as the mean ± SD, error bars represent the means of three independent experiments. $P < 0.05$

was considered statistically significant, source data are provided as a Source Data file.

Fig. S10

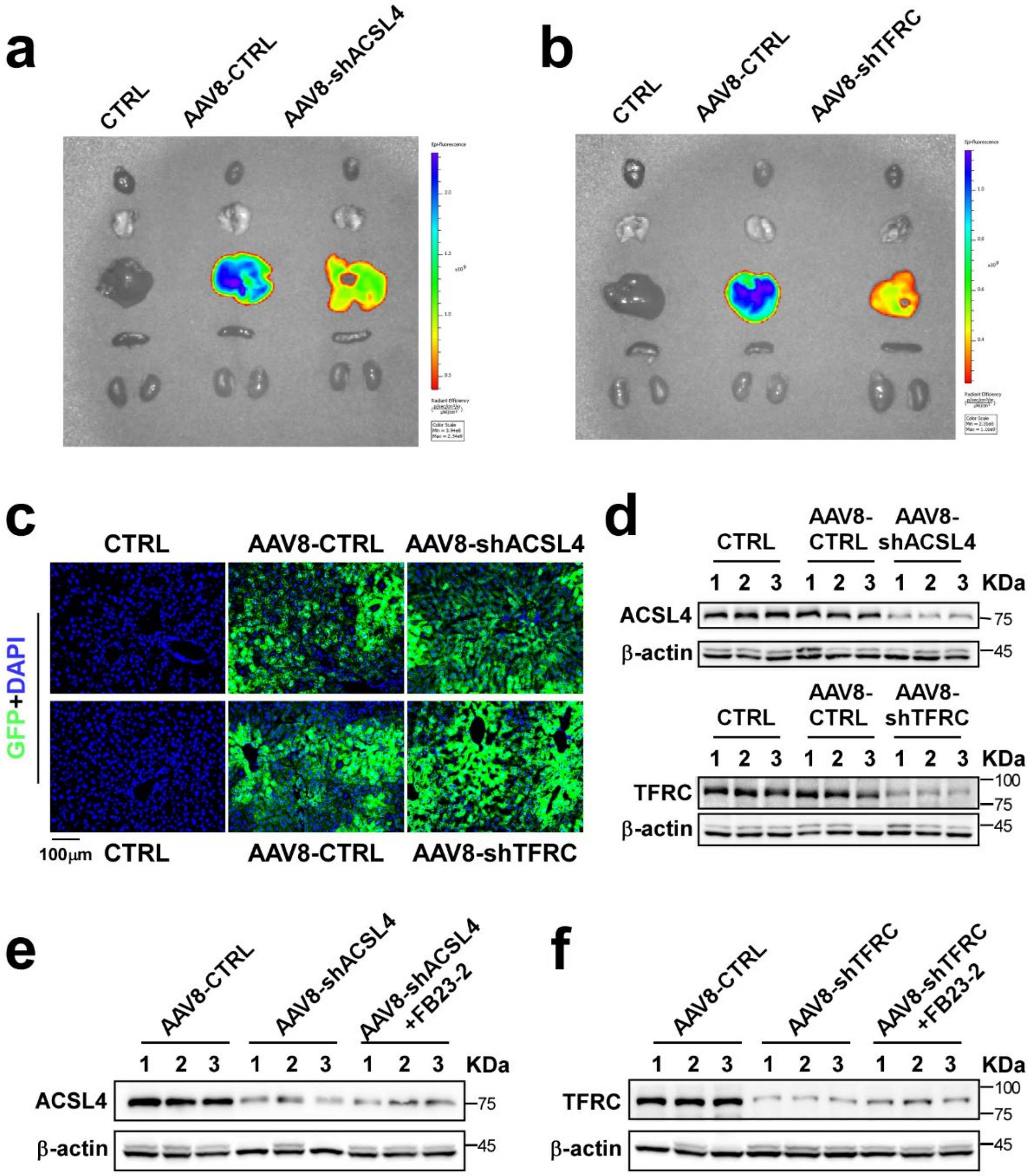

Fig. S10 Validation of the effects of AAV-shACSL4 and AAV-shTFRC. (**a-b**) Live animal imaging showed the distribution of AAVs. (**c**) Validation of the transcription and translation of AAVs in liver tissues via IF assays (magnification, 200×). (**d-f**) Western blotting showed the knockdown efficiency of relative AAVs. Three independent biological mice samples, source data are provided as a Source Data file.

Fig. S11

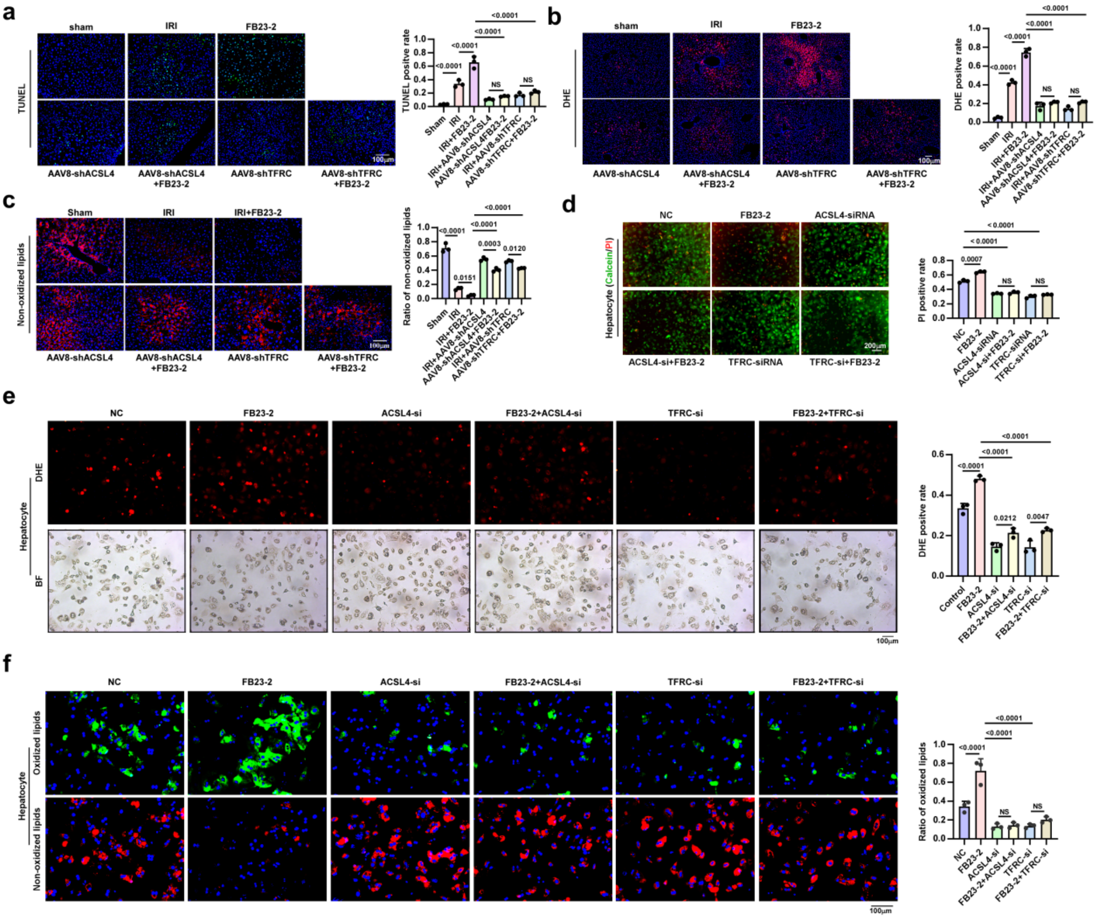

Fig. S11 FTO relies on its regulation of ACSL4 and TFRC to mitigates aged HIRI (mouse and primary hepatocytes). (**a-c**) Representative images and relative quantification of TUNEL staining (magnification, 200×), DHE staining (magnification, 100×) and C11 BODIPY staining (magnification, 200×) of the aged liver in different groups (n = 3, per group), one-way ANOVA followed by multiple comparisons. (**d-f**) Representative images and relative quantification of Calcein-AM / PI double staining assays (magnification, 100×), DHE staining (magnification, 100×) and C11 BODIPY staining (magnification, 200×) of primary hepatocytes after different treatments, one-way ANOVA followed by multiple comparisons. Statistic data are presented as the mean ± SD, error bars represent the means of three independent experiments. $P < 0.05$

was considered statistically significant. NS, no significance, source data are provided as a Source Data file.

Fig. S12

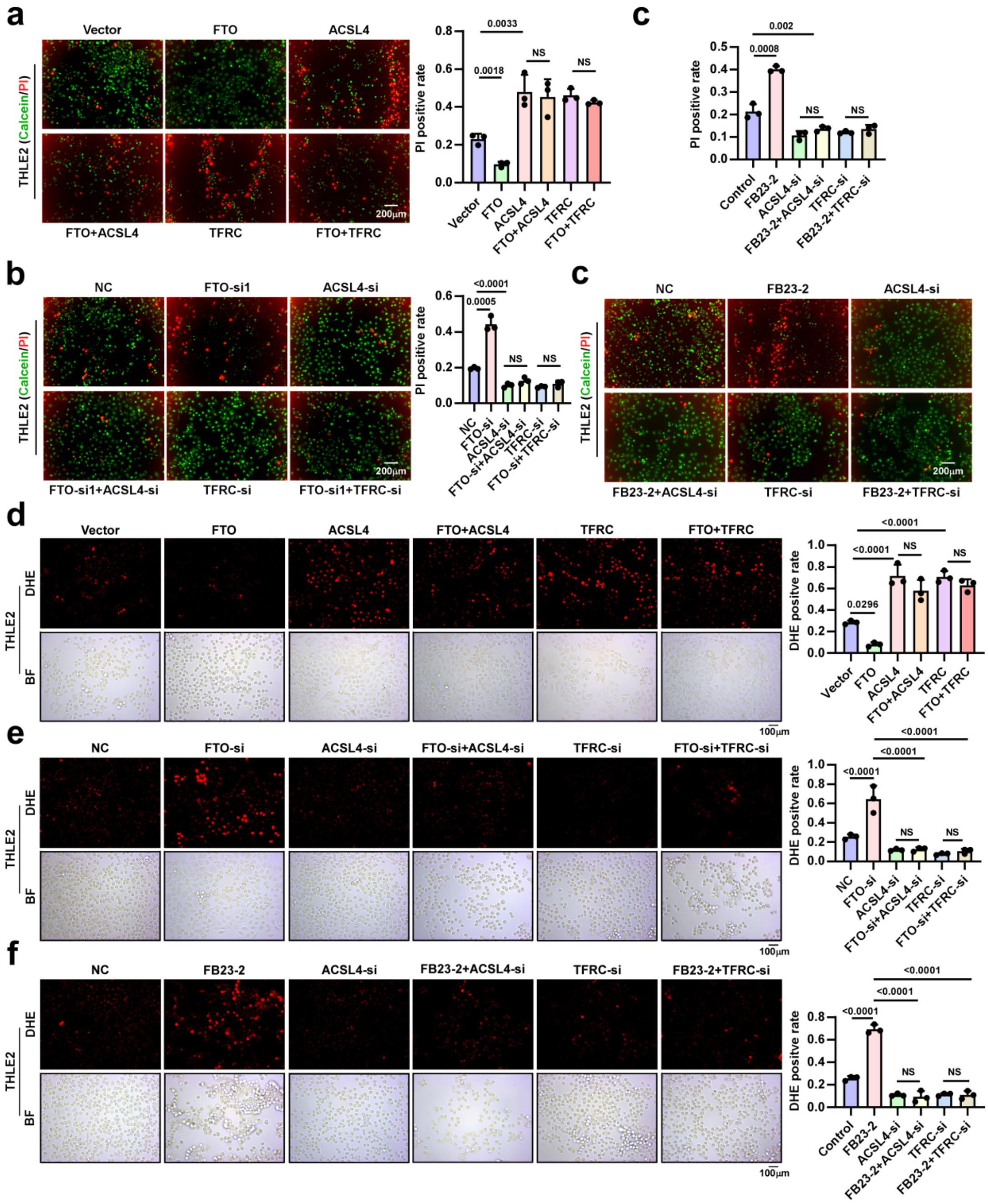

Fig. S12 FTO mitigates aged HIRI in a manner that is dependent on its regulation of ACSL4 and TFRC (THLE2 cells). (**a-c**) Representative images and relative

quantification of Calcein-AM / PI double staining assays (magnification, 100×) of senescent THLE2 cells after different treatments, one-way ANOVA followed by multiple comparisons. (**d-f**) Representative images and relative quantification of DHE staining (magnification, 100×) of senescent THLE2 cells after different treatments, one-way ANOVA followed by multiple comparisons. Statistic data are presented as the mean ± SD, error bars represent the means of three independent experiments. $P < 0.05$ was considered statistically significant. NS, no significance, source data are provided as a Source Data file.

Supplementary Tables

Table. S1 The list of differential proteins in four groups							
Y-IRI vs Y-Sham		S-IRI vs S-Sham		S-Sham vs Y-Sham		S-IRI vs Y-IRI	
Up	Down	Up	Down	Up	Down	Up	Down
Itih4	Fggy	Ppig	Ptprf	Osbpl9	Akap1	Dhtkd1	Mt1
Bin2	Cntrl	Itih4	Fggy	Mgll	Ddc	Sod3	Cd1d1
Fga	Armt1	Gls	Ttc38	Chil3	Mt1	Bhmt	Hspb1
Kng1	Etnk2	Fga	Tex264	Hsd17b7	Mup3	Cyp2j5	Gstp1
Ngp	Cyp2c23	Akap13	Aox3	F10	Cdh1	Gpld1	Sqle
Anxa3	Aox3	Kng1	Mlx	P01878	Slc7a2	Pdk4	Kpna2
Chil3	Ank3	Ngp	Casp6	H3-3a	Gstp1	Cyp1a2	Fabp5
Itgb3	Mlx	Akap1	Hgd	Apoa4	Cst3	Jchain	Sdk1
Pdk4	Slc10a1	Myadm	Ptpn9	Enpp1	Hnf1a	P01645	Rilp
F10	Sod3	Bnip3	Hpn	Fcgr2	Marcks	Igkc	Hsph1
Coro1a	Hgd	Bckdk	Gamt	Fth1	Inmt	P01843	Ugt1a9
Rbm3	Phyh	Nnmt	Aox1	H1-0	Tdo2	Igh-3	Rrp12
C3	Hpn	Gpld1	Tpmt	Il1r1	Stim2	Ighg1	Prrc2a
P01878	Gamt	Clpp	Chkb	Lgals1	Serpinf1	Ighm	Fto
H3-3a	Cyp2j5	F10	Sc5d	Rpl13a	Top1	P01878	Pwp2
Mt1	Tpmt	Afm	Idh1	Ftl1	Fabp5	P06330	Etnppl
Saa1	Diaph2	Coro1a	Dhrs3	Proc	Trmt10c	Apoa4	Tbc1d17
Saa2	Pigr	Rbm3	Cyp8b1	Rpl18	Sdk1	Enpp1	Pygb
C5	Papss2	Ca2	Adh1	Rpl28	Cobll1	Fcgr2	Hpgd
Apoa4	Ddc	C3	Dhfr	Pxmp2	Gls2	Scd1	Epm2aip1
Ptprc	Dhrs3	P01645	Ldha	H1-4	Sdf4	Lgals1	Atg5
Cfh	Cyp8b1	Igkc	Alad	Acsl4	Cbx5	Ldhb	Chchd2
Anxa2	Itm2b	P01843	Cd1d1	Aldh3a2	Ugt1a9	Hspa2	Gpx4
Serpina3k	Cyp1a2	P01864	Pfkl	Crat	Selenbp2	Slc7a2	Abhd5
Ltf	Amy1	Igh-3	Gpd1	Abcd1	Ifit1	Sdc1	Isyna1
S100a10	Col4a1	Ighg1	Slc2a2	Gck	Nostrin	Hexb	Elovl2
C4bpa	Col4a2	Hba	Glul	Sult1a1	Slc39a4	Cyp2a5	Dnajb1
Lyz2	Alad	Hbb-b1	Fasn	Fdft1	Atxn2l	Hexa	Ubqln2
Fth1	Agt	Mt1	Aldh1a1	Cda	Gvin1	Oat	
Apoa2	Scd1	Cfb	Lipc	Cyp2c37	Ppp1r10	Ldlr	
Anxa1	Slc2a2	Slc4a1	Aco1	H4c1	Ubap2l	Pxmp2	
Mpo	H2-Ab1	Saa1	Adh5	Abcc9	Fto	Acsl4	
Lcn2	Glul	Saa2	Ca8	Rpl19	Pck2	Abcd1	
Itgb2	Gstm2	P06330	Gsta3	Pla2g6	Etnppl	Slc25a4	
Apcs	Pah	C5	Fkbp4	Cd36	Larp4	Cyp2c37	
Rpl7a	Hspa2	Apoa4	Dbi	Hp1bp3	Pygb	Nrp1	
Gsn	Slc7a2	Ctsl	Proc	Uap1l1	Fermt3	Parp3	
Hspb1	Hsd3b3	Ptprc	Fah	Parp3	Ablim1	Mtus1	

Hmox1	Cebpb	Cfh	Ppm1b	Ermp1	Tat	Gbp4	
Lgals3	Ca8	Ttr	Cryz	Abcd2	Slc38a4	Lamb2	
F2	Slc6a13	Alb	Inpp1	Mme	Acmsd	Mme	
Plg	Slc6a12	Serpina1c	Ppm1a	Rpl10	Hip1	Camk2d	
Myo5b	Cyp2f2	Serpina3k	Hnf4a	Pgrmc2	Cyp4a12a	Ddx58	
Cst3	Hal	Spta1	Nat2	Pisd	F11	Tifa	
S100a8	Cd81	Ltf	Shmt1	Acot4	Atpaf2	Tbc1d5	
Grn	Ldlr	S100a10	Gss	Rint1	Cstf3	Trim14	
Ahsg	Hnf4a	Lyz2	Sqle	Tmem120a	Fars2	Plin5	
Vtn	Cyp2c37	Anxa1	Gck	Gpat4	Aasdhppt	Gpcpd1	
Hmgb2	Pcmtd1	Slc3a2	Pklr	Pitpnc1	Mrps36	Macroh2a2	
S100a9	Cdo1	Mpo	Fdft1	Tm6sf2	Mtfr1l	Ablim1	
Ptpn1	Fgf1	Lcn2	Pctp	Pex12	Arfgap3	Slc38a4	
Rpl18	Smpdl3a	Itgb2	Pitpnb	Acaa1b	Gatm	Saraf	
Abca1	Clec4f	Gsn	Tango2	Dhcr24	Slc38a3	Ifih1	
H1-4	Stim2	Ca1	Idi1	Acot11	Keg1	Ces1	
H1-1	Casp7	C1qb	Gulo	Chchd6	Slco2a1	Micu1	
Itgav	Pex7	Hmox1	Pcmtd1	Ncoa5	Lztfl1	Oasl1	
Acsl4	Rbbp6	Bpgm	Ube2d3	Fads1	Ube2j1	Cyp2c50	
Gpx3	Acp5	Sptb	Pcbd1	Tubb6	Aadat	Rab30	
Rpl6	Sult1d1	Lgals3	Ube2h	Dhrs7b	Vamp5	Dhx58	
Anxa5	Magix	Hk1	Hint1	Mgst3	Pck1	Emilin1	
Prox1	Ece1	Slc7a2	Akr1c6	Msmo1		Mrps14	
Camp	Deptor	Sdc1	Urod	Dhrs7		Chtop	
Kpna2	Tut7	F2	Khk	Tmem135		Tmem135	
Polr1c	Rilp	Cyp2a5	Pla2g6	Rpl15		Serpinb1a	
Pkm	Zzef1	Plg	Pex7	Serpinb1a		Nos1ap	
Cebpz	Lama5	Cst3	Gchfr	Lhpp		Nat8	
Cda	Lamb2	Gc	Galt	Rpl4		Ggt5	
Polr2i	Mme	Serpina1b	Acp5	Srxn1			
Rab8b	Pde3b	Marcks	Cd36	Raet1d			
Ypel5	Cmah	S100a8	Cd163	Nat8			
H4c1	Plcg1	Rpl3	Scrn3	Plscr1			
Cycs	Thrsp	Grn	Xylb	Ppl			
Supt4h1b	Selenbp2	Ahsg	Uap1l1	Stub1			
Ctps1	Rgn	Vtn	Smyd5	Vnn1			
Sptlc2	Ptprd	Saa4	Ugt3a1	Fads2			
Il6st	Cyp7a1	S100a9	C1rl	Pex11a			
G6pdx	Sat2	Serpinc1	Ece1	Ggt5			
Apoh	Mff	Rab12	Deptor				
Serpina3m	Nadsyn1	Ptpn1	Aldh16a1				
Rac2	Slc22a27	Fmr1	Rilp				
Clu	Wdr91	Abca1	Aox2				

B4galnt1	Phkb	Acsl4	Hykk				
Lsg1	Thnsl2	Gpx3	Pter				
Coq8b	Tmem106b	Epb41	Ei24				
Tsr1	Chn2	Camp	Fnta				
Orm1	Tars3	Polr1c	Cmah				
Cp	Q8BR90	Pkm	Pdcd4				
Serpinf2	Trim14	Dbt	Spr				
Arhgdib	Phka2	Polr2i	Sord				
Hp	Gprin3	Rab8b	Gstt1				
Itih3	Etnppl	Cycs	Ptprd				
Pzp	Slco2b1	Supt4h1b	Gba2				
Tgoln1	Elmo3	Mpp1	Txndc15				
Ndrg1	Gpcpd1	Vasp	Sepsecs				
Sqstm1	Asrgl1	Erh	Tatdn1				
Bcl2l1	Zhx3	Serping1	Slc44a1				
Fndc3b	Tbcel	Serpinf1	Nadsyn1				
Rrp12	Bcas3	Nrp1	Fads6				
Ptpn23	Dpyd	Sptlc2	Pgrmc2				
Wdr43	Steap3	G3bp2	Dnph1				
Fgl1	Gpat4	Bop1	Gstm7				
Slc39a14	Shtn1	Masp1	Zfyve1				
Thoc7	Afmid	G6pdx	Cpped1				
Prrc2a	Pank1	Apoa1	Fto				
Polr2e	Stradb	Serpina1d	Aldh8a1				
Lrrc8d	Tat	Serpina1e	Elovl5				
Tbl1xr1	Gpt	Gjb2	Prkaa2				
Prune1	Gga1	Apoh	Bri3bp				
Sun2	Ido2	Serpina3m	Elmo3				
Qsox1	Aldh1l1	Top1	Acat2				
Exosc6	Slc38a4	Rac2	Naprt				
Rsl1d1	Spart	Clu	Bcas3				
Tbl3	Stab1	Ssrp1	Dpyd				
Fam25c	Acmsd	Rasip1	Dnajc22				
Gnl3	Ifih1	Vars2	Hmgcs1				
Fgb	Ttc36	Trmt10c	Oplah				
Fermt3	Tlcd2	Lsg1	Gpat4				
Phldb2	Upb1	Hdgfl2	Inpp5b				
Gtf2f2	Gys2	Cln5	Afmid				
Serpina10	Cth	Elane	Pitpnc1				
Utp4	Akr1d1	Coq8b	Gpt				
Fgg	Ttc39c	Orm1	Sec14l4				
Nifk	Clec2d	Tmpo	Idnk				
Sh3bgrl3	Hnmt	Tmpo	Ido2				

Ncoa5	Dcaf11	Cybb	Apeh				
Serpina3n	Dcxr	Cfi	Tm6sf2				
Hpx	Cyp2c50	Cp	Rbks				
Lpgat1	Acy3	Pik3c2a	Fuom				
Tf	Arhgap35	Serpinf2	Ttc36				
Ddx27	Lpin1	Ecm1	Uroc1				
Tubb6	Als2	Ktn1	Tlcd2				
Nop2	Dhx58	Arhgdib	Akr1c13				
Polr2h	Emilin1	Hp	Scrn2				
Nrbp1	Mlxipl	Itih3	Gys2				
Rsl24d1	Chac2	Il1rap	Hpgd				
Gtpbp4	Cyp2u1	Mrps31	Abhd14b				
Npm3	Kynu	Pzp	Akr1d1				
Proz	Tmem53	Npm1	Alg9				
Rpl14	Lrrc57	Zyx	Ptgr2				
Golt1b	Shpk	Cavin2	Ttc39c				
Raver1	Atg7	Sqstm1	Clec2d				
Ddx28	Phyhd1	Bcl2l1	Isoc1				
Creld2	Phkg2	Fndc3b	Hnmt				
Ssbp1	Dhdh	Chd4	Dcaf11				
Rpl15	Tmprss6	Nostrin	Dcxr				
Chmp4b	Fbxo3	Wdr43	Qprt				
Srxn1	Slc38a3	Fgl1	Creld1				
Baiap2l1	Akr1e2	Slc39a14	Acy3				
Tmem43	Hsd3b7	Atxn2l	Osbpl1a				
Pak1ip1	Dpys	Ppp1r10	Dera				
Ehd4	Rapgef4	Vrk1	Grhpr				
Isyna1	Pygl	Ubap2l	Lpin1				
Nup50	Slc29a1	Tnc	Fdps				
Plscr1	Slco1b2	Nars2	Fads1				
Itga2b	Tfr2	C8b	Elovl6				
Abhd2	Msrb1	Tbl1xr1	Als2				
Dnajb1	Sult1b1	Coq3	Ormdl1				
Acot2	Slco1a1	Flna	Mthfd1				
Ubqln2	Ggcx	Pwp2	Pcyt2				
Zranb2	Mvk	Larp4	Mpi				
Acot9	Tpk1	Guf1	Bbox1				
Ppl	Hebp1	Ppfibp1	Sec14l2				
Tbk1	Ccs	Nhlrc3	Mvd				
Twf2	Pck1	C1ra	Adi1				
Rabac1		Gcc2	Stard4				
Pf4		Bclaf1	Acy1				
		Fgb	Dpp3				

		C8a	Ptdss1				
		Fermt3	Mlxipl				
		Phldb2	Hint3				
		Prpf39	Nanp				
		Cbr3	Glo1				
		Ddx18	Cuta				
		Ablim1	Chac2				
		Serpina10	Mri1				
		Slc38a4	Msmo1				
		Ctbs	Prps2				
		Pspc1	L3hypdh				
		Cant1	Pbld2				
		Fgg	Pgm3				
		Hip1	Acsl3				
		Lars2	Pgm1				
		Oasl1	Tmem53				
		Nifk	Pmvk				
		Golga4	Aacs				
		Serpina3n	Hacd2				
		Hpx	Gbe1				
		Mtif2	Lhpp				
		Atpaf2	Ggct				
		Tmlhe	Phyhd1				
		Dst	Iah1				
		Tf	Amdhd1				
		Golga2	Dhdh				
		Cyrib	Gpx4				
		Ddx27	Fbxo3				
		Chmp1a	Pbld1				
		Nop2	Aspdh				
		Kctd10	Mettl26				
		Pde2a	Stard5				
		Rab30	Hsd17b11				
		Ifitm2	Hsd3b7				
		Aass	Dpys				
		Lpin2	Pygl				
		Fdx2	Rabggta				
		Lamtor1	Raet1d				
		Mtres1	Nat8				
		Nudt8	Cml1				
		Rpl14	Slco1b2				
		Raver1	Elovl2				
		Mtfr1l	Reep6				

		Ddx28	Stard10				
		Hnrnpa0	Prep				
		Chtop	Fbp1				
		Ssbp1	Slco1a1				
		Tsfm	Ggcx				
		Gatd3a	Ndrg2				
		Bcas2	Dnajb1				
		Mrpl2	Adh4				
		Mrps9	Mvk				
		Rpl22l1	Galk1				
		Chmp4b	Nsdhl				
		Arfgap3	Pts				
		Srxn1	Hebp1				
		Exosc1	Fads2				
		Cpn2	Arih2				
		Baiap2l1	Pmm2				
		Mrps15					
		Pak1ip1					
		Ethe1					
		Wtap					
		Scamp2					
		Isyna1					
		Ddx21					
		Plscr1					
		Herpud1					
		Sars2					
		Pdk2					
		Iqgap1					
		Hip1r					
		Pnkp					
		Fetub					
		Tbl1x					
		Acot2					
		Tfrc					
		Rbpms					
		Aadat					
		Itsn2					
		Pf4					
		Hnrnpc					

Table S2. The list of primers

qRT-PCR primer (mouse)	Sequence
mACSL4-syb-up	CTCACCATTATATTGCTGCCTGT
mACSL4-syb-dn	TCTCTTTGCCATAGCGTTTTTCT
mTFRC-syb-up	ATGCCGACAATAACATGAAGGC
mTFRC-syb-dn	ACACGCTTACAATAGCCCAGG
mFTO-syb-up	CCGTCCTGCGATGATGAAGT
mFTO-syb-dn	CCCATGCCGAAATAGGGCTC
mβ-actin-syb-up	GGCTGTATTCCCCTCCATCG
mβ-actin-syb-dn	CCAGTTGGTAACAATGCCATGT
**qRT-PCR primer (human)**	
ACSL4-syb-up	CATCCCTGGAGCAGATACTCT
ACSL4-syb-dn	TCACTTAGGATTTCCCTGGTCC
TFRC-syb-up	GGCTACTTGGGCTATTGTAAAGG
TRFC-syb-dn	CAGTTTCTCCGACAACTTTCTCT
FTO-syb-up	GCTGCTTATTTCGGGACCTG
FTO-syb-dn	AGCCTGGATTACCAATGAGGA
β-actin-syb-up	CATGTACGTTGCTATCCAGGC
β-actin-syb-dn	CTCCTTAATGTCACGCACGAT
ACSL4-syb-up (meRIP)	GCTCACAGTTATTACATACACAT
ACSL4-syb-dn (meRIP)	GGAGGCAGGAACATTCAT
TFRC-syb-up (meRIP)	CACTGACCAGATAAGAATG
TRFC-syb-dn (meRIP)	AGAGTTACACCTTGGATAA
**siRNA**	
human-FTO-si1	CAGGAACCTTGGATTATAT
human-FTO-si2	GTGGCAGTGTACAGTTATA
Mouse-FTO-si1	CCTGCGATGATGAAGTGGA
Mouse-FTO-si2	GGCAGAGATCCTGATACTT
Human-ACSL4-si1	GCAGTAGTTCATGGGCTAA
Human-ACSL4-si2	CCTGCTATGGAAGCTGAAA
Human-TFRC-si1	GCGTATAGTAAGGCTGCAA
Human-TFRC-si2	GCTGGTCAGTTCGTGATTA
Mouse-ACSL4-si1	GCTGCCTGTCCACTTGTTA
Mouse-ACSL4-si2	GCATTGGGACTGAAACCAA
Mouse-TFRC-si1	CCAGATCAGCATTCTCTAA
Mouse-TRFC-si2	GCAAATGCCCAAAGCTTTA
Negative control	TTCTCCGAACGAGTCACGT
