# Supplementary material for: FTO deficiency in older livers exacerbates ferroptosis during ischaemia/reperfusion injury by upregulating ACSL4 and TFRC
Source: Nat Commun. 2024 Jun 4;15:4760. doi: 10.1038/s41467-024-49202-3 (PMC11150474; doi:10.1038/s41467-024-49202-3)
Supplement: Supplementary file 3 — Reporting Summary [file 41467_2024_49202_MOESM3_ESM.pdf]

nature portfolio

Corresponding author(s): Yingcai Zhang, Jia Yao, Jun Zheng, Yang Yang

Last updated by author(s): Jan 17, 2024

Reporting Summary

Nature Portfolio wishes to improve the reproducibility of the work that we publish. This form provides structure for consistency and transparency in reporting. For further information on Nature Portfolio policies, see our Editorial Policies and the Editorial Policy Checklist.

Statistics

For all statistical analyses, confirm that the following items are present in the figure legend, table legend, main text, or Methods section.

n/a	Confirmed	
[ ]	[x]	The exact sample size (*n*) for each experimental group/condition, given as a discrete number and unit of measurement
[ ]	[x]	A statement on whether measurements were taken from distinct samples or whether the same sample was measured repeatedly
[ ]	[x]	The statistical test(s) used AND whether they are one- or two-sided<br>*Only common tests should be described solely by name; describe more complex techniques in the Methods section.*
[x]	[ ]	A description of all covariates tested
[ ]	[x]	A description of any assumptions or corrections, such as tests of normality and adjustment for multiple comparisons
[ ]	[x]	A full description of the statistical parameters including central tendency (e.g. means) or other basic estimates (e.g. regression coefficient) AND variation (e.g. standard deviation) or associated estimates of uncertainty (e.g. confidence intervals)
[ ]	[x]	For null hypothesis testing, the test statistic (e.g. *F*, *t*, *r*) with confidence intervals, effect sizes, degrees of freedom and *P* value noted<br>*Give P values as exact values whenever suitable.*
[x]	[ ]	For Bayesian analysis, information on the choice of priors and Markov chain Monte Carlo settings
[x]	[ ]	For hierarchical and complex designs, identification of the appropriate level for tests and full reporting of outcomes
[x]	[ ]	Estimates of effect sizes (e.g. Cohen's *d*, Pearson's *r*), indicating how they were calculated

Our web collection on statistics for biologists contains articles on many of the points above.

Software and code

Policy information about availability of computer code

Data collection	Bruker Small Animal Optical Imaging System (In-Vivo XtremeII; Billerica, MA); LivingImage software 4.2(Xenogen�Alameda, CA) � A7180 488 Biochemical Analyzer (Hitachi, Japan)� light microscope (Leica, Germany); fluorescence microscope (Leica, Germany); Upright Epifluorescence (Leica, Germany); FluorChem Systems imager (ProteinSimple, CA, USA);4D-Label free Mass Spectrometer;nanoElute UHPLC system (Bruker Daltonics);TIMsTOF Promass spectrometry (Bruker Daltonics); Thermo Scientific Orbitrap Exploris 480 mass spectrometer; Nexera UHPLC LC-30A (Shimadzu)
Data analysis	GraphPad Prism 8 software (GraphPad Software, San Diego, CA); ImageJ software, USA; MaxQuant search engine(v.1.6.15.0); MaxQuant search engine (v.1.6.15.0);Venn Diagram package;InterProScan(http://www.ebi.ac.uk/interpro/) ;KEGG online service tools KEGG mapper;Wolfpsort;gplots" R-package;"Lipid Search" engine; ggplot2;scatterplot3d;Venn Diagram package;Mfuzz R package

For manuscripts utilizing custom algorithms or software that are central to the research but not yet described in published literature, software must be made available to editors and reviewers. We strongly encourage code deposition in a community repository (e.g. GitHub). See the Nature Portfolio guidelines for submitting code & software for further information.

Data

Policy information about availability of data

All manuscripts must include a data availability statement. This statement should provide the following information, where applicable:

- Accession codes, unique identifiers, or web links for publicly available datasets
- A description of any restrictions on data availability
- For clinical datasets or third party data, please ensure that the statement adheres to our policy

The MeRIP-seq raw data generated in this study have been deposited in the Genome Sequence Archive (GAS) -Human database under accession code HRA006502 [https://ngdc.cncb.ac.cn/gsa-human/browse/HRA006502]. The raw data of FTO-CLIP-seq in this study have been deposited in the Genome Sequence Archive (GAS) -Human database under accession code HRA006446 [https://ngdc.cncb.ac.cn/gsa-human/browse/HRA006446]. The mass spectrometry proteomics data have been deposited to the ProteomeXchange Consortium [https://proteomecentral.proteomexchange.org] via the iProx partner repository with the dataset identifier PXD051387 [https://www.iprox.cn/page/project.html?id=IPX0008594000]. The Lipidomics data generated in this study are provided in the Supplementary Information and Source Data file. The remaining data are available within the Article, Supplementary Information or Source Data file. Source data are provided with this paper.

Research involving human participants, their data, or biological material

Policy information about studies with human participants or human data. See also policy information about sex, gender (identity/presentation), and sexual orientation and race, ethnicity and racism.

Reporting on sex and gender	The study design did not explicitly consider sex and gender as factors. Separate data analyzes were not performed for male and female.
Reporting on race, ethnicity, or other socially relevant groupings	NO
Population characteristics	All the patients were divided into the young and old groups according to the corresponding donor age (young, age < 50; old, age >=50). Liver function evaluated by aspartate aminotransferase (AST) and alanine aminotransferase (ALT) is shown in Figure 1. For details please consult the source data.
Recruitment	We harvested liver specimens from donors of different ages before and after reperfusion during OLT at the Department of Hepatic Surgery and Liver Transplantation Center of the Third Affiliated Hospital of Sun Yat- sen University between May 2020 and November 2021. A portion of each liver sample was embedded in paraffin, and another portion was directly frozen in liquid nitrogen. Prereperfusion specimens were collected from donor livers within 3 h after cold perfusion, and reperfusion specimens were collected within 2 h after hepatic artery anastomosis.
Ethics oversight	All procedures with human samples in this study approved by the Ethics Committee of the Third Affiliated Hospital of Sun Yat-sen University (Guangzhou, China) (Approval NO. [2022]02-322-01). Informef wrtten consent was obtained from all participants aswell.

Note that full information on the approval of the study protocol must also be provided in the manuscript.

Field-specific reporting

Please select the one below that is the best fit for your research. If you are not sure, read the appropriate sections before making your selection.

☒ Life sciences ☐ Behavioural & social sciences ☐ Ecological, evolutionary & environmental sciences

For a reference copy of the document with all sections, see nature.com/documents/nr-reporting-summary-flat.pdf

Life sciences study design

All studies must disclose on these points even when the disclosure is negative.

Sample size	In vivo experiments, the sample size was determined to be enough to obtain the statistical difference between groups. For cell experiments, a minimum of three biologically independent samples was determined in this study. Sample sizes for human samples were determined based on sample availability and measurable differences across groups. Our sample sizes were chosen based on our previous experiences and to ensure adequate statistical power. Statistical methods were not used to predict sample size.
Data exclusions	No data were excluded from the analyses.
Replication	Each experiment involved at least three independent bliological replicates, as described in the figure legends. All replication attempts produced similar results.
Randomization	Mice were randomly assigned to old and young groups according to their age. For cell experiments, randomization was not necessary since each sample was analyzed equally.

Blinding

Blinding was not relevant for our animal study because the researchers needed to know the mouse age according to the experimental design. During human experiments, blinding was not possible because investigators had to document the types of diseases. During human experiments, blinding was not possible because investigators had to document the types of diseases. In cell experiments, blinding was impossible since the investigators who allocated groups also conducted treatments, processed samples, collected data, and analyzed results.

Reporting for specific materials, systems and methods

We require information from authors about some types of materials, experimental systems and methods used in many studies. Here, indicate whether each material, system or method listed is relevant to your study. If you are not sure if a list item applies to your research, read the appropriate section before selecting a response.

Materials & experimental systems

n/a	Involved in the study
☐	☒ Antibodies
☐	☒ Eukaryotic cell lines
☒	☐ Palaeontology and archaeology
☐	☒ Animals and other organisms
☐	☒ Clinical data
☒	☐ Dual use research of concern
☒	☐ Plants

Methods

n/a	Involved in the study
☒	☐ ChIP-seq
☒	☐ Flow cytometry
☒	☐ MRI-based neuroimaging

Antibodies

Antibodies used

β-Actin Mouse mAb, Cell Signaling Technology, #3700, 1:2000
FTH1 Rabbit mAb, Cell Signaling Technology, #4393, 1:2000
CISD1/mitoNEET Rabbit mAb, Cell Signaling Technology, #83775, 1:2000
NRF2 XP®Rabbit mAb, Cell Signaling Technology, #12721, 1:1000
FTO Rabbit mAb, Cell Signaling Technology, #45980, 1:1000
N6-Methyladenosine (m6A) Rabbit mAb, Cell Signaling Technology, #56593, 1:1000
Phospho-Rb (Ser780) Rabbit mAb, Cell Signaling Technology, #8180, 1:1000
Rb Mouse mAb, Cell Signaling Technology, #9309, 1:1000
Phospho-Rb (Ser807/811) XP® Rabbit mAb, Cell Signaling Technology, #8516, 1:1000
Anti-p53, Abcam, ab26, 1:1000
Anti-mouse IgG, HRP-linked Antibody, Cell Signaling Technology, #7076, 1:5000
Anti-rabbit IgG, HRP-linked Antibody, Cell Signaling Technology, #7074, 1:5000
Anti-Transferrin Receptor, Abcam, ab269513, 1:5000 (WB), 1:500 (IHC)
Anti-FACL4, Abcam, ab155282, 1:5000 (WB), 1:800 (IHC)
Anti-Glutathione Peroxidase 4, Abcam, ab125066, 1:5000
Anti-Cytokeratin 18, Abcam, ab668, 1:200 (IF)
Anti-FTO, Santa Cruz, sc-271713, 1:500 (IHC)
Anti-CDKN1B/Kip1 p27, Santa Cruz, sc-1641, 1:500
Anti-p21 Waf1/Cip1/CDKN1A, Santa Cruz, sc-6246, 1:500
NAT8 rabbit pAb, Immunoway, YN5162, 1:1000
Cy3-labeled Goat Anti-Mouse IgG (H+L), Beyotime, A0521, 1:500
DYKDDDDK Tag (D6W5B) Rabbit mAb, Cell Signaling Technology, #14793, 1:1000
Ubiquitin (P4D1) Mouse mAb, Cell Signaling Technology, #3936, 1:1000
YTHDF2 (E2I2H) Rabbit mAb, Cell Signaling Technology, #71283, 1:1000(WB), 1:100(IP)
YTHDC2 Antibody, Cell Signaling Technology, #35440, 1:1000 (WB), 1:100(IP)
Recombinant Anti-NLRP3 antibody, Abcam, ab263899, 1:1000
Gasdermin D (E9S1X) Rabbit mAb, Cell Signaling Technology, #39754, 1:1000
Recombinant Anti-pro Caspase-1 + p10 + p12 antibody, Abcam, ab179515, 1:1000(mouse)
Caspase-1 (D7F10) Rabbit mAb, Cell Signaling Technology, #3866, 1:1000 (Human)
Recombinant Anti-IL-1 beta antibody, Abcam, ab234437, 1:1000 (mouse)
IL-1β (D3U3E) Rabbit mAb, Cell Signaling Technology, #12703, 1:1000 (Human)
IL-18 (E8P5O) Rabbit mAb, Cell Signaling Technology, #57058, 1:1000 (mouse)
IL-18 (D2F3B) Rabbit mAb, Cell Signaling Technology, #54943, 1:1000 (Human)
Anti-Bcl-2 antibody, Abcam, ab196495, 1:1000
Bax Antibody, Cell Signaling Technology, #2772, 1:1000
Cleaved Caspase-3 (Asp175) (5A1E) Rabbit mAb, Cell Signaling Technology, #9664, 1:500
PARP Antibody, Cell Signaling Technology, #9542, 1:1000
RIP3 Polyclonal antibody, Proteintech, 17563-1-AP, 1:1000
MLKL Monoclonal antibody, Proteintech, 66675-1-Ig, 1:1000
Recombinant Anti-RIP3 (phospho S227) antibody, Abcam, ab209384, 1:1000(Human)
Recombinant Anti-RIP3 (phospho S232) antibody, Abcam, ab195117, 1:1000(mouse)
Recombinant Anti-MLKL (phospho S345) antibody, Abcam, ab196436, 1:1000(mouse)
Recombinant Anti-MLKL (phospho S358) antibody, Abcam, ab187091, 1:1000(Human)

Validation

All antibodies used in this study are available from commercial corporation, and have been validated by the manufacturer. Detailed

Validation

information and validation of the antibodies can be found on the manufacturer's website:
β-Actin Mouse mAb, Cell Signaling Technology, #3700,https://www.cellsignal.cn/products/primary-antibodies/b-actin-8h10d10-mouse-mab/3700
FTH1 Rabbit mAb, Cell Signaling Technology, #4393, https://www.cellsignal.cn/products/primary-antibodies/fth1-d1d4-rabbit-mab/4393
CISD1/mitoNEET Rabbit mAb, Cell Signaling Technology, #83775,https://www.cellsignal.cn/products/primary-antibodies/cisd1-mitoneet-d5m4c-rabbit-mab/83775
NRF2 XP®Rabbit mAb, Cell Signaling Technology, #12721, https://www.cellsignal.cn/products/primary-antibodies/nrf2-d1z9c-xp-rabbit-mab/12721
FTO Rabbit mAb, Cell Signaling Technology, #45980, https://www.cellsignal.cn/products/primary-antibodies/fto-d2v1i-rabbit-mab/45980
N6-Methyladenosine (m6A) Rabbit mAb, Cell Signaling Technology, #56593, https://www.cellsignal.cn/products/primary-antibodies/n6-methyladenosine-m6a-d9d9w-rabbit-mab/56593
Phospho-Rb (Ser780) Rabbit mAb, Cell Signaling Technology, #8180,https://www.cellsignal.cn/products/primary-antibodies/phospho-rb-ser780-d59b7-rabbit-mab/8180
Rb Mouse mAb, Cell Signaling Technology, #9309, https://www.cellsignal.cn/products/primary-antibodies/rb-4h1-mouse-mab/9309
Phospho-Rb (Ser807/811) XP® Rabbit mAb, Cell Signaling Technology, #8516,https://www.cellsignal.cn/products/primary-antibodies/phospho-rb-ser807-811-d20b12-xp-174-rabbit-mab/8516
Anti-mouse IgG, HRP-linked Antibody, Cell Signaling Technology, #7076, https://www.cellsignal.cn/products/secondary-antibodies/anti-mouse-igg-hrp-linked-antibody/7076
Anti-rabbit IgG, HRP-linked Antibody, Cell Signaling Technology, #7074, https://www.cellsignal.cn/products/secondary-antibodies/anti-rabbit-igg-hrp-linked-antibody/7074
DYKDDDDK Tag (D6W5B) Rabbit mAb, Cell Signaling Technology, #14793,https://www.cellsignal.cn/products/primary-antibodies/dykddddk-tag-d6w5b-rabbit-mab-binds-to-same-epitope-as-sigma-aldrich-anti-flag-m2-antibody/14793
Ubiquitin (P4D1) Mouse mAb, Cell Signaling Technology, #3936, https://www.cellsignal.cn/products/primary-antibodies/ubiquitin-p4d1-mouse-mab/3936
YTHDF2 (E2I2H) Rabbit mAb, Cell Signaling Technology, #71283, https://www.cellsignal.cn/products/primary-antibodies/ythdf2-e2i2h-rabbit-mab/71283
YTHDC2 Antibody, Cell Signaling Technology, #35440,https://www.cellsignal.cn/products/primary-antibodies/ythdc2-antibody/35440
Gasdermin D (E9S1X) Rabbit mAb, Cell Signaling Technology, #39754, https://www.cellsignal.cn/products/primary-antibodies/gasdermin-d-e9s1x-rabbit-mab/39754
Caspase-1 (D7F10) Rabbit mAb, Cell Signaling Technology, #3866, https://www.cellsignal.cn/products/primary-antibodies/caspase-1-d7f10-rabbit-mab/3866
IL-1β (D3U3E) Rabbit mAb, Cell Signaling Technology, #12703, https://www.cellsignal.cn/products/primary-antibodies/il-1b-d3u3e-rabbit-mab/12703
IL-18 (E8P5O) Rabbit mAb, Cell Signaling Technology, #57058, https://www.cellsignal.cn/products/primary-antibodies/il-18-e8p5o-rabbit-mab/57058
IL-18 (D2F3B) Rabbit mAb, Cell Signaling Technology, #54943,https://www.cellsignal.cn/products/primary-antibodies/il-18-d2f3b-rabbit-mab/54943
Bax Antibody, Cell Signaling Technology, #2772, https://www.cellsignal.cn/products/primary-antibodies/bax-antibody/2772
Cleaved Caspase-3 (Asp175) (5A1E) Rabbit mAb, Cell Signaling Technology, #9664, https://www.cellsignal.cn/products/primary-antibodies/cleaved-caspase-3-asp175-5a1e-rabbit-mab/9664
PARP Antibody, Cell Signaling Technology, #9542, https://www.cellsignal.cn/products/primary-antibodies/parp-antibody/9542
Anti-Transferrin Receptor, Abcam, ab269513,https://www.abcam.cn/products/primary-antibodies/transferrin-receptor-antibody-h684-ab269513.html
Anti-p53, Abcam, ab26, https://www.abcam.cn/products/primary-antibodies/p53-antibody-pab-240-ab26.html
Anti-FACL4, Abcam, ab155282, https://www.abcam.cn/products/primary-antibodies/facl4-antibody-epr8640-ab155282.html
Anti-Glutathione Peroxidase 4, Abcam, ab125066, https://www.abcam.cn/products/primary-antibodies/glutathione-peroxidase-4-antibody-epncir144-ab125066.html
Anti-Cytokeratin 18, Abcam, ab668, https://www.abcam.cn/products/primary-antibodies/cytokeratin-18-antibody-c-04-ab668.html
Recombinant Anti-NLRP3 antibody, Abcam, ab263899,https://www.abcam.cn/products/primary-antibodies/nlrp3-antibody-epr23094-1-ab263899.html
Recombinant Anti-pro Caspase-1 + p10 + p12 antibody, Abcam, ab179515, https://www.abcam.cn/products/primary-antibodies/pro-caspase-1--p10--p12-antibody-epr16883-ab179515.html
Recombinant Anti-IL-1 beta antibody, Abcam, ab234437, https://www.abcam.cn/products/primary-antibodies/il-1-beta-antibody-epr16805-15-ab234437.html
Anti-Bcl-2 antibody, Abcam, ab196495, https://www.abcam.cn/products/primary-antibodies/bcl-2-antibody-ab196495.html
Recombinant Anti-RIP3 (phospho S227) antibody, Abcam, ab209384, https://www.abcam.cn/products/primary-antibodies/rip3-phospho-s227-antibody-epr9627-ab209384.html
Recombinant Anti-RIP3 (phospho S232) antibody, Abcam, ab195117, https://www.abcam.cn/products/primary-antibodies/rip3-phospho-s232-antibody-epr9516n-25-ab195117.html
Recombinant Anti-MLKL (phospho S345) antibody, Abcam, ab196436, https://www.abcam.cn/products/primary-antibodies/mlkl-phospho-s345-antibody-epr95152-ab196436.html
Recombinant Anti-MLKL (phospho S358) antibody, Abcam, ab187091, https://www.abcam.cn/products/primary-antibodies/mlkl-phospho-s358-antibody-epr9514-ab187091.html
Anti-FTO, Santa Cruz, sc-271713, https://www.scbt.com/p/fatso-antibody-c-3?requestFrom=search
Anti-CDKN1B/Kip1 p27, Santa Cruz, sc-1641, https://www.scbt.com/p/p27-antibody-f-8?requestFrom=search
Anti-p21 Waf1/Cip1/CDKN1A, Santa Cruz, sc-6246, https://www.scbt.com/p/p21-antibody-f-5?requestFrom=search
NAT8 rabbit pAb, Immunoway, YN5162, http://www.immunoway.com/Home/Search?keywords=YN5162
Cy3-labeled Goat Anti-Mouse IgG (H+L), Beyotime, A052https://www.beyotime.com/product/A0521.htm
RIP3 Polyclonal antibody, Proteintech, 17563-1-AP, https://www.ptgcn.com/products/RIPK3-Antibody-17563-1-AP.htm
MLKL Monoclonal antibody, Proteintech, 66675-1-Ig, https://www.ptgcn.com/products/MLKL-Antibody-66675-1-Ig.htm

Eukaryotic cell lines

Policy information about cell lines and Sex and Gender in Research

Cell line source(s)	Primary hepatocytes were isolated from young and old male mice, THLE2 (JNO-CRL-2706) were purchased from GuangZhou Jennio Biotech Co.,Lt .
Authentication	STR testing.
Mycoplasma contamination	The mycoplasma contamination of all cell lines was negative .
Commonly misidentified lines (See ICLAC register)	This study did not use any misidentified cell lines.

Animals and other research organisms

Policy information about studies involving animals; ARRIVE guidelines recommended for reporting animal research, and Sex and Gender in Research

Laboratory animals	The animals used in this experiment were all mice on the C57BL/6 background, and young mice (10 weeks old, male) and old mice (20 months old, male) were purchased from the Model Animal Research Center of Nanjing University (Nanjing, China). All the experimental mice were housed in a specific pathogen-free (SPF) environment with a temperature of 25°C, a humidity of 40%-70%, and a 12 h light-dark cycle. The animal procedures were carried out in compliance with Chinese legislation regarding the use of experimental animals and approved by the Institutional Animal Care and Use Committee (ACUC), Jennio Biotech Co., Ltd. The grouping of each part of the experiment followed the principle of randomization.
Wild animals	No wild animals were used in this study.
Reporting on sex	In this study, all animals were males, and there were no sex-based analyses .
Field-collected samples	No sample was collected from the field.
Ethics oversight	All experimental procedures involving animals were carried out complying with the Chinese legislation regarding experimental animals and approved by the Institutional Animal Care and Use Committee (IACUC), Jennio Biotech Co., Ltd. (Approval No. JENNIO-IACUC-2022-A003).

Note that full information on the approval of the study protocol must also be provided in the manuscript.

Clinical data

Policy information about clinical studies

All manuscripts should comply with the ICMJE guidelines for publication of clinical research and a completed CONSORT checklist must be included with all submissions.

Clinical trial registration	N/A
Study protocol	N/A
Data collection	N/A
Outcomes	N/A
